# Massive variceal bleeding secondary to splenic vein thrombosis successfully treated with splenic artery embolization: a case report

**DOI:** 10.1186/1752-1947-4-139

**Published:** 2010-05-19

**Authors:** Daniel Paramythiotis, Theodossis S Papavramidis, Konstantinos Giavroglou, Stamatia Potsi, Fotis Girtovitis, Antonis Michalopoulos, Vassilis N Papadopoulos, John Prousalidis

**Affiliations:** 1 First Propedeutic Department of Surgery, AHEPA University Hospital, Aristotle University of Thessaloniki, Thessaloniki, Greece; 2 Department of Radiology, AHEPA University Hospital, Aristotle University of Thessaloniki, Thessaloniki, Greece; 3 First Propedeutic Department of Internal Medicine, AHEPA University Hospital, Aristotle University of Thessaloniki, Thessaloniki, Greece

## Abstract

**Introduction:**

Splenic vein thrombosis results in localized portal hypertension called sinistral portal hypertension, which may also lead to massive upper gastrointestinal bleeding. Symptomatic sinistral portal hypertension is usually best treated by splenectomy, but interventional radiological techniques are safe and effective alternatives in the management of a massive hemorrhage, particularly in cases that have a high surgical risk.

**Case presentation:**

We describe a 23-year-old Greek man with acute massive gastric variceal bleeding caused by splenic vein thrombosis due to a missing von Leiden factor, which was successfully managed with splenic arterial embolization.

**Conclusions:**

Interventional radiological techniques are attractive alternatives for patients with a high surgical risk or in cases when the immediate surgical excision of the spleen is technically difficult. Additionally, surgery is not always successful because of the presence of numerous portal collaterals and adhesion. Splenic artery embolization is now emerging as a safe and effective alternative to surgery in the management of massive hemorrhage from gastric varices due to splenic vein thrombosis, which often occurs in patients with hypercoagulability.

## Introduction

Massive gastrointestinal bleeding may result either from esophageal (gastric varices) or from portal hypertensive gastropathy. Sinistral portal hypertension (SPH) is a clinical syndrome of gastric variceal hemorrhage in the setting of splenic vein thrombosis (SVT), mostly due to pancreatic pathology [1]. Unlike patients with generalized portal hypertension, most patients with SVT are usually asymptomatic and have a normal hepatic function. Bleeding from gastric varices (GVs) is generally more severe than from esophageal varices [2], although it occurs less frequently [3-5].

The diagnosis of SPH is achieved by a combination of gastroscopy, liver function tests, ultrasound examination and/or contrast-enhanced computed tomography (CT) scan of the abdomen [1].

Splenectomy is considered the treatment of choice for splenic vein thrombosis complicated by variceal hemorrhage or hypersplenism (symptomatic). On the other hand, embolotherapy has a great spectrum of clinical applications such as the following: (i) trauma, (ii) tumors, (iii) infertility among men, (iv) impotence, and (v) vascular malformations [6]. It is a novelty to apply embolotherapy to SPH.

We describe a patient with massive gastric variceal bleeding caused by SPH. His SPH was due to blood hypercoagulability and attributed to a missing von Leiden factor (FVL). He was successfully treated by splenic arterial embolization.

## Case presentation

A 23-year-old Greek man who had episodes of hematemesis and hematochezia was admitted to the emergency department of our hospital. Clinical signs of anemia and splenomegaly were recognized on our patient. His medical history revealed that he was missing an FVL. His peripheral blood examinations revealed the following: hematocrit, 22.2%; hemoglobin, 7.5 g/dL; international normalized ration (INR), 1.22. Results of his liver function tests, as well as the rest of his biochemical examinations, were within normal limits.

An emergency endoscopy performed on our patient showed enlarged bleeding gastric varices but no esophageal varices. This led us to consider that the enlarged varices may be secondary to splenic vein thrombosis. We used a Sengstaken-Blakemore tube on our patient, but this failed to restrain his bleeding.

**Figure 1:**
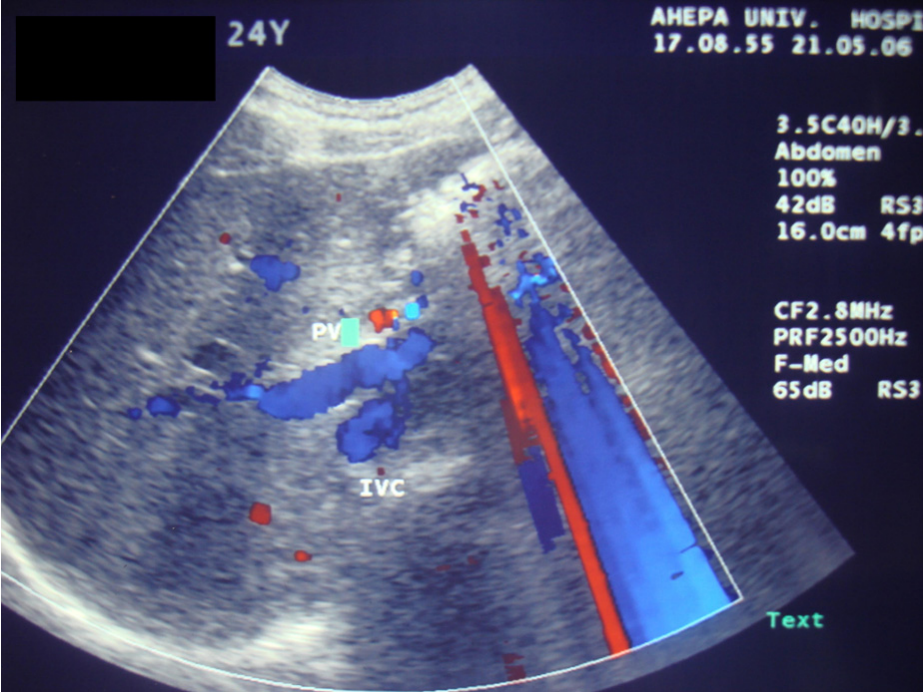
**Ultrasonography showing dilated splenic vein of our patient**.

**Figure 2:**
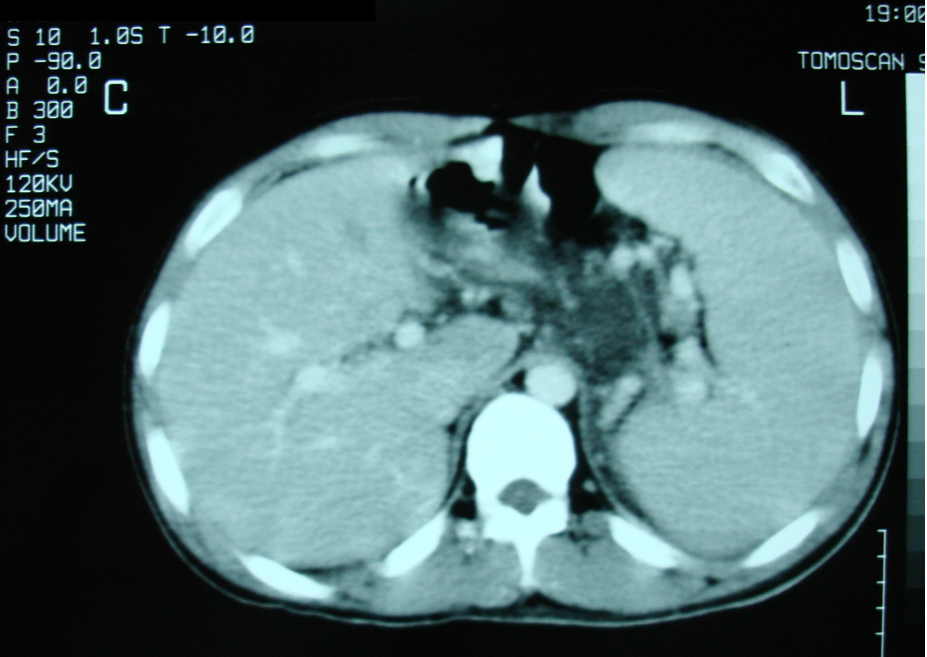
**Computed tomography scan presenting an enlarged spleen**.

Ultrasound and CT scan of our patient revealed his enlarged spleen and an engorged splenic artery with a diameter of 1 cm, and a fusiform dilated splenic vein measuring 5 × 6 × 9 cm (Figures 1 and 2).

An angiography was performed using the Seldinger technique on the femoral artery of our patient. Selective celiac angiography and superselective splenic arteriography with frontal and bilateral oblique projections were also performed. A venous phase follow-up examination demonstrated that our patient had a completely occluded splenic vein. GVs and dilated gastroepiploic veins were also noted.

**Figure 3:**
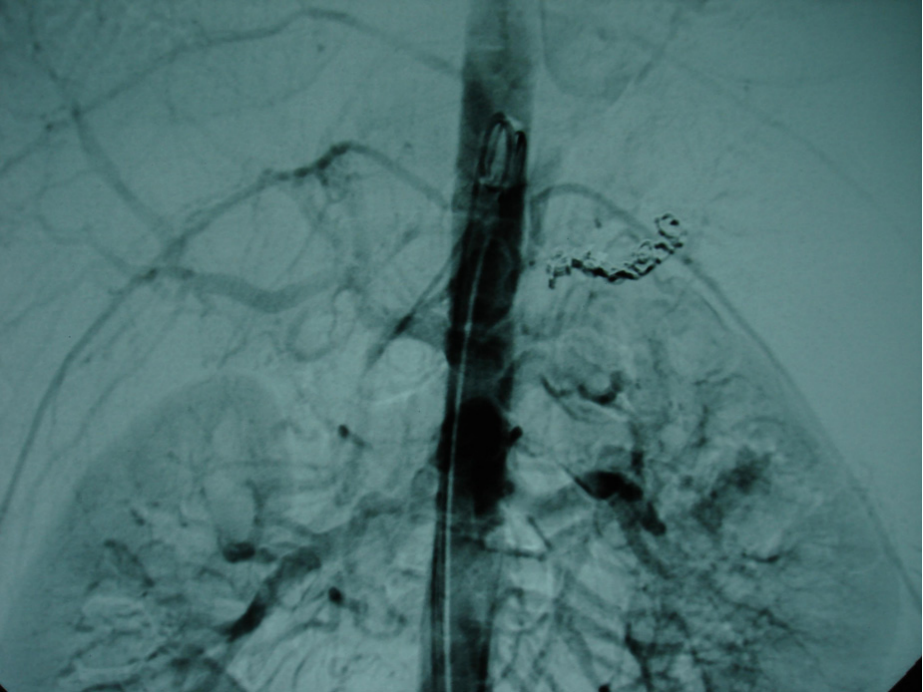
**Angiography showing catheterization and embolization of the splenic artery using Gianturco steel coils**.

An emergency embolization of our patient's splenic artery was subsequently performed. A guidewire was directed into his splenic artery, and a wedge balloon catheter was passed over the guidewire using several giant Gianturco steel coils. Initially, a coil with a diameter of 10 mm and a length of 10 cm was used because his splenic artery measured around 10 mm in diameter on CT images. Subsequently, smaller coils (5 mm × 5 cm) were used to occlude the lumen of the 10 mm × 10 cm coil (Figure 3). The procedure was uneventful and the bleeding of his GVs eventually subdued.

Our patient was discharged one week later without any symptoms. An elective surgical splenectomy was also scheduled.

## Discussion

FVL deficiency has been reported in 2% to 30% of patients with portal vein thrombosis [7]. This wide variation makes it difficult to assess the importance of FVL as a predisposing factor [7]. Koshy *et al. *found that FVL was also highly associated with splenic vein thrombosis [8]. It is hardly surprising, therefore, to find SVT in patients with FVL deficiency, such in the case of our patient.

Regardless of the pathogenesis, splenic vein thrombosis leads to a localized sinistral venous hypertension which causes the splenic venous outflow to return via low-pressure collaterals, thus preventing the circulation of blood from the spleen [9]. Pathways via the short gastric and/or gastro-epiploic veins cause dilatation of the sub-mucosal venous system in the stomach and esophagus. This is coupled with the formation of thin-walled gastric and esophageal varices [9,10]. Because blood drainage is diverted by the coronary vein to the portal system, the presence of gastric varices without esophageal varices is a very specific sign of splenic vein occlusion. In the case we report here emergency gastroscopy revealed the sole existence of bleeding gastric varices.

Splenic vein thrombosis may be either symptomatic or asymptomatic. Gastrointestinal bleeding at varying severity (anemia, hematemesis, melena, or hematochezia) is the most common manifestation of this syndrome [9-12]. In a study by Sakorafas et al., gastrointestinal bleeding complicated splenic vein thrombosis in 18% of our patients they reported, although splenomegaly was a constant finding in all patients [13]. In our case, the varices of our patient were symptomatic and presented with hematemesis.

Prophylactic splenectomy to prevent gastric variceal hemorrhage has been recommended for patients with splenic vein thrombosis, but the benefit of splenectomy is difficult to determine. Moreover, different treatment options for gastric variceal bleeding secondary to splenic vein thrombosis have been proposed. Splenectomy was formerly considered the best treatment [11,14,15]. Endoscopic injection sclerotherapy in patients with GVs is more difficult to perform than when esophageal varices are involved [16]. Meanwhile, portal systemic shunting is not indicated because of normal portal pressure and hepatic function. Partial splenic arterial embolization, which reduces blood flow through the spleen, is considered an effective alternative treatment.

## Conclusions

Interventional radiological techniques are attractive alternatives for patients with a high surgical risk or in cases when immediate surgical excision of the spleen is technically difficult and is sometimes unsuccessful because of the presence of numerous portal collaterals and adhesion. Splenic artery embolization is now emerging as a safe and effective alternative to surgery in the management of massive hemorrhage from gastric varices due to splenic vein thrombosis, which is not a rare condition in patients with hypercoagulability.

## Consent

Written informed consent was obtained from our patient for publication of this case report and any accompanying images. A copy of the written consent is available for review by the Editor-in-Chief of this journal.

## Competing interests

The authors declare that they have no competing interests.

## Authors' contributions

DP, FG and TSP received our patient in the emergency department, analyzed and interpreted his data, and drafted the manuscript. AM, VNP and JP were the surgeons involved, edited the manuscript, and were the treating doctors. SP and KG were the radiologists involved. All authors read and approved the final manuscript.
